# The Role of Micro-Communities in Post-Disaster Psychological Well-Being of Older Adults: A Cross-Sectional Study

**DOI:** 10.3390/bs16040503

**Published:** 2026-03-27

**Authors:** Selman Bolukbasi

**Affiliations:** Department of Gerontology, Faculty of Health Sciences, Inonu University, 44280 Malatya, Türkiye; selman.bolukbasi@inonu.edu.tr; Tel.: +90-5069590051

**Keywords:** aged, family support, disasters, psychological well-being, social support

## Abstract

Background: Older adults are highly vulnerable to adverse psychological outcomes following large-scale disasters. Social micro-communities are often assumed to play a protective role in post-disaster recovery. This study examined the association between perceived micro-community support and psychological outcomes among older adults after the 2023 earthquakes in Malatya, Türkiye. Methods: This cross-sectional study included 287 community-dwelling adults aged 60 years and older from the Battalgazi and Yesilyurt districts. Data were collected through face-to-face interviews using a sociodemographic form, the Multidimensional Scale of Perceived Social Support, the Warwick–Edinburgh Mental Well-Being Scale, and the Satisfaction with Life Scale. Non-parametric statistical analyses were applied. Results: Younger participants reported significantly higher perceived social support and psychological well-being (*p* < 0.05). Male and married participants demonstrated greater life satisfaction (*p* < 0.05). Educational status was significantly associated with family support and total perceived social support (*p* < 0.05). Although most participants perceived micro-communities as important, perceived importance was not significantly associated with psychological well-being or life satisfaction. Health problems and economic hardship were the most common post-disaster stressors. Conclusions: Informal social support alone may be insufficient to promote psychological recovery among disaster-affected older adults. Targeted community-based interventions addressing health and socioeconomic needs are required.

## 1. Introduction

Disasters represent complex events that profoundly disrupt social, economic, and psychological systems, with older adults being among the most vulnerable populations affected (Yavuz et al., 2024). Age-related physical frailty, chronic health conditions, heightened risk of social isolation, and cognitive decline substantially increase older adults’ susceptibility during post-disaster recovery processes (Ay & Cetin, 2022; Gencer, 2020). Evidence from previous disasters, including the 2007 earthquake in Peru, indicates that older adults’ disaster experiences are shaped by gender norms, family dynamics, and sociocultural contexts (Shenk et al., 2010).

Social support derived from micro-communities, such as family members, neighbors, and community-based organizations, has been widely recognized as a key protective factor for older adults in disaster settings (Sevim, 2024; Akyildiz et al., 2018). Research conducted after the 2011 Great East Japan Earthquake demonstrated that community-based initiatives involving older adults contributed to improved social cohesion at both family and neighborhood levels (Lee et al., 2022). These micro-community structures have been associated with enhanced psychosocial resilience and reduced risks of adverse outcomes, including loneliness, depressive symptoms, and cognitive decline among older populations (Hikichi et al., 2020; Sasaki et al., 2020).

In this study, micro-communities are conceptualized as small-scale, geographically proximate social networks that include family members, neighbors, and community-based organizations providing instrumental, emotional, and informational support in daily life. This conceptualization draws on Coleman’s (1988) social capital framework and Aldrich’s (2012) work on community resilience in disaster contexts, which emphasize that recovery depends not only on macro-level resources but also on localized bonding and bridging social capital. The term “micro-communities” is used to highlight the scale and embeddedness of these support structures, distinguishing them from broader conceptualizations of “social networks” or “community,” which may not capture the proximal, face-to-face interactions central to older adults’ everyday experiences (Coleman, 1988; Aldrich, 2012).

Despite their potential benefits, micro-community support mechanisms may be weakened by post-disaster conditions such as forced displacement, resettlement, disruption of social networks, and limited access to resources (Akcakaya & Suzuki Him, 2021; Hashimoto et al., 2015; Ozgun-Basibuyuk et al., 2021). While emotional support, social integration, and a sense of belonging are central elements of recovery, the effectiveness of micro-communities may vary depending on contextual and structural factors within disaster-affected environments (Yotsui et al., 2016; Greiner et al., 2016).

In parallel, sociodemographic characteristics including age, gender, education, and income level have consistently been shown to influence health-related outcomes in older adults following disasters (Shenk et al., 2010; Kwak et al., 2017; De Luca et al., 2022; Toguc, 2025). These findings highlight the importance of considering individual and social determinants when evaluating post-disaster recovery processes and planning health and social support services for aging populations.

Although the international literature on disaster recovery and aging is expanding, empirical field-based evidence examining the role of micro-communities in post-disaster recovery among older adults in Türkiye remains limited. The province of Malatya, which experienced extensive physical destruction and social disruption following the 2023 earthquakes, provides a critical context for examining local solidarity structures and recovery dynamics. Micro-level analyses conducted in this setting may yield valuable insights into the functioning of community-based support mechanisms and the resilience capacities of older adults.

While previous studies have examined social support broadly in disaster contexts (Lee et al., 2022; Hikichi et al., 2020; Sasaki et al., 2020), the present study advances the existing literature in three key ways. First, it conceptualizes and examines micro-communities (family members, neighbors, and community-based organizations) as distinct analytical units rather than treating social support as a monolithic construct, providing more granular insight into how proximal support networks function in disaster-affected settings. Second, it empirically tests the assumption that the perceived importance of micro-communities automatically translates into better psychological outcomes—an association that is often implied in the literature but insufficiently examined empirically. Third, it offers context-specific evidence from Türkiye’s 2023 earthquakes, where traditional communal solidarity structures coexist with rapid urbanization and institutional gaps, thereby providing a valuable comparative lens for understanding how micro-community support mechanisms operate in non-Western disaster contexts with distinct sociocultural dynamics.

Accordingly, this study aimed to examine the association between perceived social support from micro-communities and psychological well-being, life satisfaction, and health-related outcomes among individuals aged 60 years and above living in the Battalgazi and Yesilyurt districts of Malatya. A secondary objective was to generate context-specific implications to inform post-disaster social support strategies and community-based interventions targeting older adults.

## 2. Materials and Methods

### 2.1. Study Design and Setting

This study was designed as a cross-sectional, explanatory field study conducted to examine the association between perceived support from micro-communities and psychological well-being, life satisfaction, and health satisfaction among older adults following a disaster. The research was carried out in the Battalgazi and Yesilyurt districts of Malatya province, Turkiye, which were severely affected by the 2023 earthquakes.

### 2.2. Participants and Sampling

The study population consisted of community-dwelling individuals aged 60 years and above residing in the Battalgazi and Yesilyurt districts. Due to post-earthquake displacement and institutional disruption, no pre-existing sampling frame was available. Therefore, a systematic intercept sampling approach was employed in public spaces frequently used by older adults, including parks, gardens, and community centers in both districts. These locations enabled access to older adults who remained mobile and engaged in daily social activities, which was essential for assessing functional and psychosocial recovery. Door-to-door sampling was not feasible due to ongoing housing instability and safety concerns.

Data were collected through face-to-face questionnaire interviews conducted by the author, a researcher specialized in geropsychiatry with professional experience working with older adults. Trauma-informed communication principles were applied throughout the data collection process, and particular care was taken to ensure confidentiality, voluntary participation, and psychological safety. Participants showing signs of acute psychological distress were informed about available local mental health services.

Age (60 years and above) was the primary inclusion criterion. Geographic stratification by district (Battalgazi and Yesilyurt) was applied to ensure representation, but no quotas were imposed for gender, disaster exposure, or socioeconomic characteristics. The resulting sample distribution (65.5% male, 80.1% married) reflects the demographic profile of older adults who remained community-dwelling and socially active in the post-disaster context and is acknowledged as a potential source of sampling bias.

According to 2023 Turkish Statistical Institute data, the combined population aged 60 years and above in the Battalgazi and Yesilyurt districts was approximately 23,450 individuals. Using a 95% confidence level, a 5% margin of error, and assuming a 50% population proportion (a conservative estimate for maximum variability), the calculated minimum sample size was 378 participants. However, given post-disaster constraints including displacement, mortality, and limited accessibility, a target of 287 participants was set to maintain statistical adequacy while acknowledging feasibility limitations. The final sample included 139 participants from Battalgazi District and 148 from Yesilyurt District, representing approximately 1.2% of the target population. While this sampling fraction is statistically adequate for inferential purposes, it may not capture the full diversity of post-disaster experiences, particularly among the most vulnerable older adults who were hospitalized, severely injured, relocated outside the districts, or otherwise unable to participate in community-based activities.

To ensure confidentiality in public settings, interviews were conducted in semi-private areas (e.g., quiet corners of parks, secluded benches, or designated rooms in community centers) away from other individuals. Participants were informed of their right to decline any question or terminate the interview at any time without consequence. All interviews were conducted by the researcher using standardized questionnaire administration procedures. Because all interviews were conducted by a single trained researcher, interrater reliability was not applicable. To assess data entry accuracy, a random sample of 10% of completed questionnaires (*n* = 29) was independently reviewed and verified, yielding a concordance rate of 98.5%. While some participants may have experienced prior disasters, no screening for previous disaster exposure was conducted, as the analytical focus of the study was explicitly limited to recovery experiences following the February 2023 earthquakes, which were directly experienced by all participants residing in the study districts.

### 2.3. Data Collection Instruments

**Figure 1:**
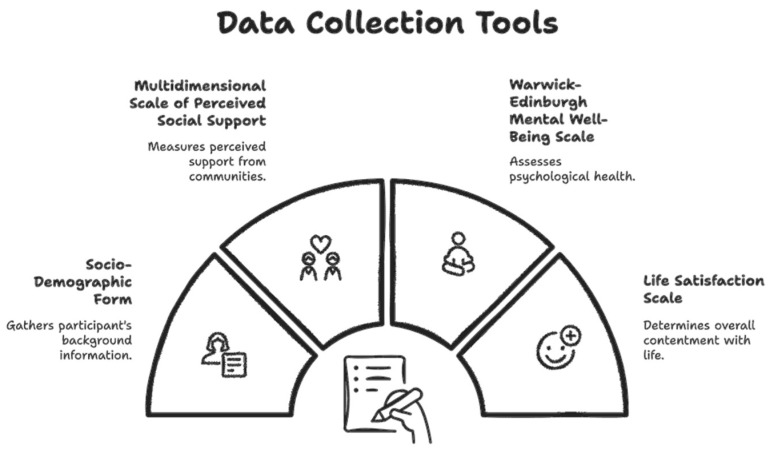
Data Collection Tools.

Data were collected using a structured questionnaire comprising the following instruments:

(i). A Sociodemographic Information Form developed by the researchers;
(ii). The Multidimensional Scale of Perceived Social Support (MSPSS);
(iii). The Warwick–Edinburgh Mental Well-being Scale (WEMWBS); and
(iv). The Satisfaction with Life Scale (SWLS) (Figure 1).

#### 2.3.1. Multidimensional Scale of Perceived Social Support

The Multidimensional Scale of Perceived Social Support (MSPSS) is a 12-item self-report instrument designed to assess perceived social support from three sources: family, friends, and a significant other. Each subscale consists of four items rated on a 7-point Likert scale, with higher scores indicating greater perceived social support (Zimet et al., 1988).

The Turkish adaptation of the scale, revised by Eker, Arkar, and Yaldız, included culturally appropriate modifications to enhance conceptual clarity. In this adaptation, “family” was defined to include parents, spouse, children, and siblings, while “significant other” referred to individuals outside the family and friendship network, such as neighbors or healthcare professionals. The Turkish version demonstrated high internal consistency (Cronbach’s α ranging from 0.80 to 0.95) and confirmed the original three-factor structure (Eker et al., 2001).

#### 2.3.2. Warwick–Edinburgh Mental Well-Being Scale

The Warwick–Edinburgh Mental Well-being Scale (WEMWBS) is a 14-item self-report scale developed to measure positive mental health and psychological well-being in the general population. Items reflect both hedonic and eudaimonic aspects of well-being and are rated on a 5-point Likert scale ranging from “none of the time” to “all of the time,” with higher scores indicating greater mental well-being (Tennant et al., 2007).

The Turkish validation study conducted by Keldal confirmed the unidimensional structure of the scale and reported high internal consistency (Cronbach’s α = 0.92), supporting its suitability for population-based research and well-being assessments (Keldal, 2015).

#### 2.3.3. Satisfaction with Life Scale

The Satisfaction with Life Scale (SWLS) was developed by Diener et al. to assess individuals’ global cognitive judgments of life satisfaction. The scale consists of five items rated on a Likert-type scale, yielding total scores ranging from 5 to 25, with higher scores indicating greater life satisfaction. No reverse-coded items are included (Diener et al., 1985).

The Turkish adaptation by Dagli and Baysal demonstrated strong psychometric properties (Cronbach’s α = 0.88; test–retest reliability r = 0.97), supporting its internal consistency and construct validity and indicating that the scale is suitable for both research and applied settings (Dagli & Baysal, 2016).

### 2.4. Inclusion and Exclusion Criteria

Inclusion criteria were: being 60 years of age or older; having experienced a disaster (e.g., earthquake, flood, or fire) within the past five years; residing in the Battalgazi or Yesilyurt districts; providing informed consent; and completing the questionnaire in full. Individuals who did not meet these criteria were excluded from the study.

### 2.5. Ethical Considerations

All participants were informed about the purpose and procedures of the study, and written informed consent was obtained. Ethical approval was granted by the Inonu University Social and Human Sciences Scientific Research and Publication Ethics Committee in accordance with the Declaration of Helsinki (Ref. No: 2025/14-6).

### 2.6. Statistical Analysis

Statistical analyses were conducted using IBM SPSS Statistics version 26.0 for macOS (IBM Corp., Armonk, NY, USA). Descriptive statistics were reported as mean, standard deviation, frequency, and percentage. Data normality was assessed using skewness and kurtosis values, which indicated non-normal distribution. Therefore, non-parametric statistical tests were employed, including the Mann–Whitney U test, Kruskal–Wallis test, chi-square test, and Spearman’s rank correlation analysis. Statistical significance was set at *p* < 0.05.

## 3. Results

A total of 287 older adults aged 60 years and above participated in the study. The mean age was 63.8 ± 3.8 years (range: 60–83 years), with a median of 63 years. The majority of participants were male (*n* = 188, 65.5%) and married (*n* = 230, 80.1%). Participants were almost equally distributed between the Yesilyurt (*n* = 148, 51.6%) and Battalgazi (*n* = 139, 48.4%) districts.

Regarding educational level, most participants were literate without formal schooling (*n* = 107, 37.3%) or had completed primary education (*n* = 98, 34.1%). A smaller proportion had secondary school (*n* = 17, 5.9%), high school (*n* = 29, 10.1%), or higher education (*n* = 25, 8.7%). Most participants lived in extended family structures (*n* = 186, 64.8%) and were homeowners (*n* = 236, 82.2%). The mean household size was 2.52 ± 1.30 persons, and 13.9% of participants lived alone.

Economically, the vast majority reported that their income was insufficient to meet expenses (*n* = 275, 95.8%). Health-related difficulties were the most frequently reported post-disaster challenge (*n* = 173, 60.3%), followed by the need for psychological support (*n* = 59, 20.6%) and housing problems (*n* = 34, 11.8%).

During the recovery process, participants most frequently reported receiving support from non-governmental organizations (*n* = 114, 39.7%) and neighbors (*n* = 100, 34.8%), followed by family members (*n* = 40, 13.9%) and local government (*n* = 24, 8.4%). Nine participants (3.1%) reported receiving no support.

Regarding the perceived role of micro-communities, 47.0% of participants rated them as important and 44.9% as very important. Only a small proportion rated their role as moderate or less important.

The mean total MSPSS score was 72.0 ± 9.7. Subscale scores were 23.2 ± 4.5 for family support, 24.5 ± 3.3 for friend support, and 24.3 ± 3.1 for significant other support. The mean WEMWBS score was 38.4 ± 4.9, indicating moderate psychological well-being. The mean SWLS score was 16.8 ± 4.2, reflecting moderate life satisfaction.

Significant gender differences were observed in life satisfaction scores, with males reporting higher SWLS scores than females (17.3 ± 4.3 vs. 16.1 ± 3.9; Z = −2.37, *p* = 0.02). No significant gender differences were found for MSPSS subscales or WEMWBS scores.

Participants younger than 63 years reported significantly higher family support, friend support, significant other support, and total MSPSS scores compared to those aged 63 years and above (*p* < 0.05). No significant age-related differences were found for WEMWBS or SWLS scores.

Educational level was significantly associated with family support (H = 15.32, *p* = 0.009) and total MSPSS scores (H = 14.07, *p* = 0.02). Post hoc analyses indicated that participants with secondary school education reported higher family and total social support scores compared to those with high school education.

Married participants reported lower family and friend support scores than single participants (*p* < 0.05). However, married participants demonstrated significantly higher psychological well-being scores on the WEMWBS (38.6 ± 4.8 vs. 37.2 ± 4.9; Z = −2.05, *p* = 0.04).

Significant differences in WEMWBS scores were observed based on the primary source of support during recovery (H = 9.51, *p* = 0.05). Participants who received support from local government exhibited the highest mental well-being scores, whereas those who reported receiving no support had the lowest scores.

Strong positive correlations were observed among MSPSS subscales and total scores. Family support was strongly correlated with friend support (r = 0.881, *p* < 0.001) and total MSPSS scores (r = 0.923, *p* < 0.001). Friend support showed a strong correlation with total MSPSS scores (r = 0.928, *p* < 0.001). A moderate positive correlation was found between psychological well-being and life satisfaction (r = 0.495, *p* < 0.001). Additionally, a weak positive correlation was observed between significant other support and life satisfaction (r = 0.130, *p* = 0.027) (Table 1).

## 4. Discussion

This study examined the role of micro-communities in the post-disaster recovery of older adults living in the Battalgazi and Yesilyurt districts of Malatya following the 2023 earthquakes, with a focus on perceived social support, psychological well-being, and life satisfaction. The findings indicate that sociodemographic characteristics, particularly age, gender, education, and marital status, are more strongly associated with psychosocial outcomes than the perceived importance of micro-communities themselves.

Younger participants reported higher levels of perceived social support across all MSPSS subscales and demonstrated better psychological well-being compared to older participants. This finding is consistent with previous research suggesting that younger older adults tend to have broader and more active social networks and greater functional capacity to engage in social interactions following disasters (Shenk et al., 2010; Hikichi et al., 2020). Age-related declines in social engagement and increasing health-related limitations may reduce access to support resources among older age groups, thereby diminishing the protective effects of social networks in post-disaster contexts.

Gender differences emerged only in life satisfaction, with male participants reporting higher levels than females. This finding aligns with studies highlighting the gendered nature of disaster recovery, where women may experience disproportionate emotional, caregiving, and domestic burdens after disasters (Shenk et al., 2010). However, the absence of gender differences in perceived social support and psychological well-being contrasts with some previous findings (Kwak et al., 2017), suggesting that cultural and familial structures in Malatya may buffer gender-based disparities in access to social support.

Educational level was associated with perceived family support and total social support, with participants who had completed secondary education reporting higher support levels than those with higher education. While higher education is generally associated with greater social capital, this finding may reflect the importance of close-knit family and neighborhood ties among individuals with lower educational attainment in this setting. In post-disaster environments, informal support networks may outweigh professional or institutional resources, particularly in regions characterized by strong communal bonds.

Marital status was another significant factor influencing psychosocial outcomes. Single participants reported higher perceived family and friend support, whereas married participants demonstrated higher psychological well-being. This pattern suggests that marriage may provide emotional stability and continuity that enhances well-being, while single individuals may compensate for the absence of a spouse by relying more heavily on extended family and social networks. Similar patterns have been observed in disaster-related studies emphasizing the stabilizing role of spousal support (Sevim, 2024; Akyildiz et al., 2018).

Despite the high proportion of participants who rated micro-communities as important or very important, perceived importance was not significantly associated with social support, psychological well-being, or life satisfaction scores. This finding contrasts with intervention-based studies demonstrating the effectiveness of structured micro-community initiatives in post-disaster recovery (Lee et al., 2022). One possible explanation is the broad conceptualization of micro-communities in the present study, which encompassed family members, neighbors, and non-governmental organizations. Such heterogeneity may have obscured the differential effects of specific support sources. Moreover, shared trauma within families and communities may limit the emotional benefits of support in the immediate post-disaster period.

The weak associations observed between perceived social support and psychological well-being or life satisfaction suggest that social support alone may be insufficient to offset the cumulative impact of post-disaster stressors. High rates of health problems, housing difficulties, and income inadequacy among participants may have constrained the potential benefits of micro-community support. These findings are consistent with previous research emphasizing the moderating role of socioeconomic and health-related factors in post-disaster recovery processes (Akcakaya & Suzuki Him, 2021; Greiner et al., 2016).

Several descriptive patterns merit further consideration. First, the relatively small proportion of participants reporting the loss of relatives (10.8%) likely reflects sampling constraints rather than low mortality in the affected region. Community-dwelling older adults who experienced multiple bereavements or severe trauma may have been institutionalized, relocated outside the district, or unable to participate due to psychological or physical limitations. Consequently, the present findings may underestimate the psychological burden among the most severely affected older adults.

Second, support was reported more frequently from non-governmental organizations (39.7%) and neighbors (34.8%) than from government sources (8.4%). This distribution reflects both the rapid mobilization of civil society in Turkiye’s disaster response context and possible gaps in formal service delivery, particularly in peripheral neighborhoods. While this pattern illustrates community resilience, it also raises concerns about sustainability, as NGO involvement is often temporary and neighbor capacity may become strained when entire communities share similar trauma and resource constraints.

Third, the absence of significant differences by residential location (city center versus areas distant from the city) suggests that the earthquake’s impact was geographically widespread rather than spatially concentrated. This interpretation is consistent with documented structural damage across both Battalgazi and Yesilyurt districts.

No significant differences were observed across several structural and contextual variables, including place of residence, housing type, household composition, income status, and chronic disease presence. This may indicate a homogenizing effect of the disaster, whereby shared exposure and collective adversity reduce variability in recovery outcomes. Nevertheless, the high prevalence of health-related problems and the reported need for psychological support underscore the ongoing vulnerability of older adults in disaster-affected settings.

Several limitations should be acknowledged. The cross-sectional design precludes causal interpretations, and reliance on self-reported data may introduce response bias. Longitudinal studies are needed to examine changes in social support and psychosocial outcomes over time. In addition, qualitative research could provide deeper insight into how different types of micro-community support operate and how older adults perceive and utilize these resources. Overall, the findings suggest that while micro-communities are culturally valued and widely recognized, their effectiveness in promoting psychological well-being and life satisfaction may depend on broader structural and contextual conditions. Strengthening formal support mechanisms and addressing health, housing, and economic challenges should therefore be central components of post-disaster recovery strategies for older adults.

## 5. Conclusions

This study investigated the role of social micro-communities in the post-disaster recovery of adults aged 60 years and older living in the Battalgazi and Yesilyurt districts of Malatya following the 2023 earthquakes. The findings indicate that sociodemographic characteristics, particularly age, gender, education, and marital status, are significantly associated with perceived social support, psychological well-being, and life satisfaction. Younger participants demonstrated higher levels of perceived social support and psychological well-being, while married and male individuals reported greater life satisfaction.

Despite the high cultural value attributed to micro-communities, their perceived importance was not directly associated with psychological well-being or life satisfaction. This suggests that, in post-disaster contexts, informal social support alone may be insufficient to counterbalance the cumulative effects of health-related problems, housing instability, and economic hardship commonly experienced by older adults.

These findings highlight the need for strengthening structured, accessible, and context-sensitive community-based support mechanisms that complement informal social networks. Integrating psychosocial services with health, housing, and social welfare interventions may be essential to enhance recovery and promote long-term well-being among disaster-affected older populations.

Several methodological and theoretical directions warrant further investigation. Longitudinal designs are needed to examine how the role and potential protective effects of micro-communities change across different phases of disaster recovery, particularly as formal assistance decreases and informal networks reorganize. Qualitative and mixed-methods approaches could provide deeper insight into how various forms of micro-community support, including family members, neighbors, and non-governmental organizations, are experienced and interpreted by older adults within specific cultural contexts.

In addition, the absence of gender differences in perceived social support, despite differences in life satisfaction, calls for comparative research across cultural and disaster settings to clarify when and how social and familial structures may buffer or amplify gender-related vulnerabilities. Finally, intervention-oriented studies are needed to test integrated models that combine formal services with community-based support. Such research would help determine whether strengthening micro-community capacity leads to measurable improvements in psychological outcomes, or whether broader structural interventions, such as income, housing, and healthcare support, play a more decisive role in post-disaster recovery among older adults.

## Figures and Tables

**Table 1:** Sociodemographic characteristics and psychosocial outcomes of participants.

Sociodemographic Status				MSPSS				WEMWBS	SWLS
Features		N	%	Family	Friend	Significant Other	Total		
**Total**		287	100						
**Gender**	Male	188	65.5	23.4 ± 4.4	24.6 ± 3.3	24.3 ± 3.08	72.2 ± 9.5	38.3 ± 4.9	*17.3 ± 4.3*
	Female	99	34.5	22.9 ± 4.8	24.4 ± 3.9	24.3 ± 3	71.6 ± 10	38.5 ± 4.8	*16.1 ± 3.9*
				Z = −0.91 *p* = 0.37	Z = −0.14 *p* = 0.89	Z = −0.06 *p* = 0.95	Z = −0.26 *p* = 0.80	Z = −0.37 *p* = 0.71	*Z* = *−2.37* *p* = *0.02*
**Age**	Mean ± SD	63.8 ± 3.8 (60–83)	Below 63 (139)	23.8 ± 4.4	24.9 ± 3.2	24.8 ± 2.8	73.6 ± 9.2	38.6 ± 4.5	16.9 ± 4.2
	Median/Mode	63/60	63 and above (148)	22.7 ± 4.6	24.1 ± 3.4	23.8 ± 3.3	70.6 ± 9.9	38.2 ± 5.2	16.8 ± 4.2
				*Z* = *−2.43* *p* = *0.02*	*Z* = *−2.33* *p* = *0.02*	*Z* = *−2.56* *p* = *0.01*	*Z* = *−2.79* *p* = *0.005*	Z = −0.45 *p* = 0.65	Z = −0.16 *p* = 0.88
**Education**	Illiterate	11	3.8	23.4 ± 4.1	24.5 ± 3.1	24 ± 3.2	71.9 ± 9.3	38.5 ± 4.6	16.5 ± 3.7
	Literate	107	5.9	22.7 ± 5.5	23.8 ± 4	23.8 ± 3.2	70.3 ± 11.1	38 ± 6.3	17 ± 5.1
	Primary School	98	34.1	23.04 ± 4.4	24.6 ± 3.3	24.4 ± 2.9	72.1 ± 9.8	38.1 ± 4.7	17 ± 4.5
	Secondary School	17	5.9	25 ± 5	25.4 ± 4.2	25.7 ± 3.2	76.1 ± 11.8	37.7 ± 4.5	16.8 ± 4
	High School	29	10.1	20.2 ± 4.4	23 ± 3.2	24.1 ± 3.1	67.2 ± 7.4	39.9 ± 5.4	18.3 ± 5
	Higher Education and above	25	8.7	23.2 ± 4.5	25.6 ± 2.6	25.1 ± 3	75.8 ± 7.5	38.5 ± 4.5	16.8 ± 3.3
				H = 15.32 *p* = *0.009*	H = 8.76 *p* = 0.12	H = 7.65 *p* = 0.18	*H* = *14.07* *p* = *0.02*	H = 3.15 *p* = 0.68	H = 3.15 *p* = 0.68
**Marital** **Status**	Single	57	19.9	24.5 ± 3.6	25.5 ± 2.7	24.5 ± 3	74.5 ± 8.2	37.2 ± 4.9	16 ± 3.9
	Married	230	80.1	22.9 ± 4.7	24.3 ± 3.4	24.2 ± 3.1	71.4 ± 9.9	38.6 ± 4.8	17.1 ± 4.6
				Z = *−2.45* *p* = *0.01*	*Z* = *−2.35* *p* = *0.02*	Z = −0.38 *p* = 0.70	Z = −0.26 *p* = 0.80	*Z* = *−2.05* *p* = *0.04*	*Z* = *−1.70* *p* = *0.09*
**Place of** **Residence**	Battalgazi	139	48.4	23.2 ± 4.7	24.6 ± 3.4	24.5 ± 3.1	72.2 ± 10.2	38.6 ± 4.7	17.2 ± 4.2
	Yesilyurt	148	51.6	23.2 ± 4.4	24.5 ± 3.2	24.1 ± 3.6	71.8 ± 9.2	38.1 ± 5	16.9 ± 4.2
				Z = −0.36 *p* = 0.72	Z = 0.38 *p* = 0.71	Z = −1.04 *p* = 0.30	Z = −0.77 *p* = 0.44	Z = −0.87 *p* = 0.38	Z = −1.51 *p* = 0.13
**Residence Type**	Apartment	144	50.2	23.6 ± 4.2	24.7 ± 3.2	24.6 ± 3	72.8 ± 9.1	38.4 ± 5.1	16.8 ± 4.1
	Detached	143	49.8	22.9 ± 4.8	24.4 ± 3.4	24 ± 3.1	71.2 ± 10.1	38.3 ± 4.6	16.9 ± 4.3
				Z = −1.03 *p* = 0.30	Z = −0.83 *p* = 0.41	Z = −1.63 *p* = 0.10	Z = −1.33 *p* = 0.18	Z = 0.62 *p* = 0.53	Z = 0.06 *p* = 0.95
**Family Type**	Nuclear Family	101	35.2	*24 ± 3.9*	24.9 ± 2.9	24.2 ± 3.1	73.1 ± 8.7	38.1 ± 5	16.7 ± 4.1
	Extended Family	186	64.8	*22.8 ± 4.8*	24.3 ± 3.5	24.3 ± 3	71.4 ± 10.1	38.5 ± 4.8	17 ± 4.3
				*Z* = *−1.75* *p* = *0.08*	Z = −1.0 *p* = 0.32	Z = −0.15 *p* = 0.88	Z = −1.10 *p* = 0.28	Z = −0.46 *p* = 0.65	Z = −0.41 *p* = 0.68
**Property Ownership**	Homeowner	236	82.2	23 ± 4.6	24.3 ± 3	24.3 ± 3	71.7 ± 9.7	38.4 ± 5	16.8 ± 4.2
	Tenant	51	17.8	24.1 ± 4.1	24.2 ± 3.3	24.2 ± 3.3	73.3 ± 9.7	37 ± 4.2	17.5 ± 4.1
				Z = −1.56 *p* = 0.12	Z = −1.02 *p* = 0.31	Z = −0.02 *p* = 1.0	Z = −1.14 *p* = 0.25	Z = −0.71 *p* = 0.48	Z = −0.98 *p* = 0.33
**Number of residents**	Mean ± SD	2.52 ± 1.30 (1–9)	Alone (40)	22.5 ± 5.9	23.7 ± 4.1	24.3 ± 3.4	70.5 ± 12.3	38 ± 4.9	17.4 ± 4.5
	Median/Mode	2/2	With someone (247)	23.3 ± 4.3	24.6 ± 3.2	24.3 ± 3	72.3 ± 9.2	38.4 ± 4.9	16.8 ± 4.1
				Z = −0.19 *p* = 0.85	Z = −0.93 *p* = 0.35	Z = −0.27 *p* = 0.79	Z = −0.38 *p* = 0.75	Z = −0.51 *p* = 0.61	Z = −0.65 *p* = 0.52
**House** **Location**	Downtown	54	18.8	23.3 ± 4.1	24.4 ± 3.2	24.9 ± 2.8	72.6 ± 9.7	37.9 ± 5.4	16.7 ± 4.2
	Close to the City	114	39.7	22 ± 4.8	24.2 ± 3.4	24 ± 3.1	70.8 ± 10.1	38.8 ± 5.1	16.8 ± 4.2
	Distant from the City	119	41.5	23.2 ± 4.5	24.9 ± 3.2	24.4 ± 3.1	73 ± 9.1	38.1 ± 4.3	17.1 ± 4.2
				H = 2.05 *p* = 0.36	H = 1.90 *p* = 0.39	H = 4.25 *p* = 0.12	H = 2.81 *p* = 0.25	H = 2.07 *p* = 0.35	H = 0.64 *p* = 0.73
**Income** **Status**	Income is less than expenses	275	95.8	23.1 ± 4.6	24.4 ± 3.4	24.3 ± 3.1	71.8 ± 9.7	38.4 ± 4.9	16.9 ± 4.2
	Income equals expenses	12	4.2	25.3 ± 3.3	26.3 ± 2.6	24.7 ± 2.7	76.3 ± 7	37.4 ± 4.5	15.6 ± 4.3
				Z = −1.50 *p* = 0.13	*Z* = *−2.17* *p* = *0.30*	Z = −0.35 *p* = 0.73	Z = −1.50 *p* = 0.13	Z = −0.55 *p* = 0.58	Z = −1.34 *p* = 0.18
**Loss of Relatives After Disaster**	Yes	31	10.8	24.1 ± 3.7	25.2 ± 2.7	25.1 ± 2.4	74.4 ± 7.5	38.4 ± 4.7	17.3 ± 4.2
	No	256	89.2	23.1 ± 4.6	24.4 ± 3.4	24.2 ± 2.1	71.7 ± 9.9	38.4 ± 4.9	16.8 ± 4.2
				Z = −0.95 *p* = 0.34	Z = −1.06 *p* = 0.31	Z = −1.30 *p* = 0.20	Z = −1.20 *p* = 0.24	Z = −0.04 *p* = 0.97	Z = 0.25 *p* = 0.80
**Post-Disaster Difficulties**	Housing Problem	34	11.8	23.4 ± 5.2	24.2 ± 4	24 ± 3.7	71.7 ± 11.6	39.2 ± 4.8	16.7 ± 4.5
	Access to Food and Basic Needs	6	2.1	25.7 ± 4.3	25.8 ± 3.1	24.5 ± 3.4	76 ± 8.94	35.5 ± 3.5	15.3 ± 2.6
	Health Problems	173	60.3	23 ± 4.7	24.6 ± 3.3	24.2 ± 3	71.8 ± 9.7	38.3 ± 5.1	16.7 ± 4.2
	Psychological Support	59	20.6	23.4 ± 4	24.5 ± 3	24.9 ± 3	72.7 ± 8.9	38.3 ± 4.7	17.7 ± 4.1
	Social Isolation	15	5.2	23.2 ± 3.5	24.3 ± 3.3	23.4 ± 2.8	70.9 ± 7.9	38.8 ± 4.3	17 ± 3.7
				H = 3.16 *p* = 0.53	H = 1.88 *p* = 0.76	H = 4.52 *p* = 0.34	H = 1.86 *p* = 0.76	H = 3.77 *p* = 0.44	H = 4.0 *p* = 0.41
**Support During the** **Recovery Process**	Family Members	40	13.9	21.7 ± 4.8	23.5 ± 3.6	23.4 ± 2.9	68.6 ± 10	38.1 ± 3.6	17.5 ± 3.4
	Neighbors	100	34.8	23.5 ± 4.5	24.6 ± 3.4	24.4 ± 3.1	72.4 ± 9.8	37.7 ± 5.4	16.7 ± 4.2
	Local Government	24	8.4	23.8 ± 3.7	24.7 ± 2.5	24.1 ± 2.8	72.6 ± 7.9	40.3 ± 3.5	17 ± 3.1
	NGOs	114	39.7	23.3 ± 4.5	24.7 ± 3.2	24.5 ± 3.1	72.5 ± 9.5	38.8 ± 4.9	16.9 ± 4.7
	Unsupported	9	3.1	24.67 ± 4.8	25.67 ± 3.2	24.67 ± 3.71	75 ± 11.36	35.78 ± 4.21	15.6 ± 2.7
				H = 5.74 *p* = 0.22	H = 5.13 *p* = 0.28	H = 6.19 *p* = 0.19	H = 6.92 *p* = 0.14	*H* = *9.51* *p* = *0.05*	H = 2.55 *p* = 0.64
**The Role of Micro-** **Communities**	Insignificant	5	1.7	20.8 ± 4.8	21.8 ± 4.7	22 ± 3.5	64.6 ± 11.2	40.2 ± 4	17.4 ± 4.2
	Less important	4	1.4	23.3 ± 8.9	24.5 ± 5.7	24.8 ± 3.2	72.5 ± 16.5	35.3 ± 5.9	14 ± 0.8
	Moderate	14	4.9	22.8 ± 4.6	24.2 ± 3.8	24.3 ± 3.4	71.3 ± 10.9	37.9 ± 4.4	16.6 ± 4.6
	Important	135	47	23.4 ± 4.5	24.7 ± 3.3	24.3 ± 3	72.4 ± 9.7	37.7 ± 5.1	16.4 ± 4.2
	Very Important	129	44.9	23.1 ± 4.5	24.2 ± 3.8	24.4 ± 3.1	72 ± 9.2	35.3 ± 5.9	17.5 ± 4.1
				H = 2.99 *p* = 0.56	H = 3.27 *p* = 0.51	H = 2.84 *p* = 0.59	H = 3.39 *p* = 0.50	*H* = *8.21* *p* = *0.08*	H = 6.31 *p* = 0.18
**Health Status**	Very Dissatisfied	32	11.1	21.6 ± 5.2	23.3 ± 3.8	23.6 ± 2.8	68.5 ± 10.5	38 ± 4.7	16.4 ± 4.5
	Dissatisfied	62	21.6	23.6 ± 4.4	25 ± 3.4	24.5 ± 3.2	73 ± 9.9	39.2 ± 4.4	17.1 ± 4.1
	Moderate	36	12.5	23.1 ± 4.6	24.5 ± 3.2	24.3 ± 2.9	71.9 ± 9.1	37.9 ± 4.9	17.2 ± 4.7
	Satisfied	146	50.9	23.5 ± 4.2	24.7 ± 3.1	24.3 ± 3.1	72.5 ± 9.1	38.3 ± 5	16.8 ± 4.1
	Very Satisfied	11	3.8	22.7 ± 6.4	23.8 ± 4.4	24.4 ± 3.2	70.9 ± 13.1	37.6 ± 6.3	17.3 ± 3.7
				H = 3.77 *p* = 0.44	H = 5.74 *p* = 0.22	H = 3.07 *p* = 0.55	H = 4.98 *p* = 0.29	H = 3.08 *p* = 0.54	H = 0.60 *p* = 0.96
**Psychological Status**	Very bad	7	2.4	23.7 ± 4.2	25 ± 3	26 ± 2.2	74.7 ± 8.4	36.6 ± 3.2	15 ± 3.5
	Bad	38	13.2	21.5 ± 5	24.4 ± 3.8	23.2 ± 3.4	68.1 ± 10.9	38.7 ± 4.9	16.6 ± 3.8
	Normal	50	17.4	23.6 ± 4.5	24.6 ± 3.3	24.2 ± 3.1	72.5 ± 9.8	39.1 ± 5	17.7 ± 4.4
	Good	181	63.1	23.5 ± 4.4	24.8 ± 3.2	24.5 ± 3	72.7 ± 9.3	38.2 ± 4.9	16.9 ± 4.3
	Very Good	11	3.8	22.4 ± 4.4	23.4 ± 3.2	23.8 ± 3.4	69.5 ± 9.7	37.6 ± 4	15.9 ± 2.4
				H = 5.87 *p* = 0.21	H = 6.15 *p* = 0.19	H = 7.60 *p* = 0.11	H = 7.42 *p* = 0.12	H = 3.73 *p* = 0.44	H = 3.70 *p* = 0.45
**Chronic** **Disease**	Yes	201	70	23.2 ± 4.5	24.5 ± 3.3	24.4 ± 3	72.1 ± 9.5	38.6 ± 4.6	17 ± 4.2
	No	86	30	23.3 ± 4.6	24.6 ± 3.5	24 ± 3.3	71.8 ± 10	37.8 ± 5.4	16.5 ± 4.1
				Z = −0.30 *p* = 0.76	Z = −0.41 *p* = 0.68	Z = −0.65 *p* = 0.52	Z = −0.03 *p* = 0.98	Z = −1.22 *p* = 0.22	Z = −0.82 *p* = 0.41

Z = Mann–Whitney Test. H = Kruskal–Wallis Test.

## Data Availability

The data presented in this study are available on reasonable request from the corresponding author. The data are not publicly available due to ethical and privacy restrictions involving human participants.

## Abbreviations

The following abbreviations are used in this manuscript:

MSPSS	Multidimensional Scale of Perceived Social Support
WEMWBS	Warwick–Edinburgh Mental Well-Being Scale
SWLS	Satisfaction with Life Scale
NGO	Non-Governmental Organization
SD	Standard Deviation

## References

[B1-behavsci-16-00503] Akcakaya E. Y., Suzuki Him M. (2021). Yaşlıların sosyal ağlarının afet riski bağlamında değerlendirilmesi. Senex Yaşlılık Çalışmaları Dergisi.

[B2-behavsci-16-00503] Akyildiz N. A., Gurboga S., Gurboga C. (2018). Yaşlı afetzedelerin geçici barınma ihtiyaçlarının karşılanması üzerine örnek bir çalışma: Kahramanmaraş-Elbistan prefabrik huzurevi kompleksi. Sosyal Politika Çalışmaları Dergisi.

[B3-behavsci-16-00503] Aldrich D. P. (2012). Building resilience: Social capital in post-disaster recovery..

[B4-behavsci-16-00503] Ay S., Cetin B. (2022). Deprem mağduru yaşlı bireylerin mekan memnuniyetlerinin incelenmesi: Elazığ örneği. Sosyolojik Bağlam Dergisi.

[B5-behavsci-16-00503] Coleman J. S. (1988). Social capital in the creation of human capital. American Journal of Sociology.

[B6-behavsci-16-00503] Dagli A., Baysal N. (2016). Adaptation of the satisfaction with life scale into Turkish. Electronic Journal of Social Sciences.

[B7-behavsci-16-00503] De Luca V., Femminella G. D., Patalano R., Formosa V., Lorusso G., Rivetta C., Di Lullo F., Mercurio L., Rea T., Salvatore E., Korkmaz Yaylagul N., Apostolo J., Silva R. C., Dantas C., van Staalduinen W. H., Liotta G., Iaccarino G., Triassi M., Illario M. (2022). Assessment tools of biopsychosocial frailty dimensions in community-dwelling older adults. International Journal of Environmental Research and Public Health.

[B8-behavsci-16-00503] Diener E., Emmons R. A., Larsen R. J., Griffin S. (1985). The satisfaction with life scale. Journal of Personality Assessment.

[B9-behavsci-16-00503] Eker D., Arkar H., Yaldiz H. (2001). Çok Boyutlu Algılanan Sosyal Destek Ölçeği’nin Gözden Geçirilmiş Formunun Faktör Yapısı, Geçerlik ve Güvenirliği. Turk Psikiyatri Dergisi.

[B10-behavsci-16-00503] Gencer N. (2020). COVID-19 sürecinde yaşlı olmak: 65 yaş ve üstü vatandaşlar için uygulanan sokağa çıkma yasağı üzerine değerlendirmeler ve manevi sosyal hizmet. Türkiye Sosyal Hizmet Araştırmaları Dergisi.

[B11-behavsci-16-00503] Greiner C., Ono K., Otoguro C., Chiba K., Ota N. (2016). Intervention for the maintenance and improvement of physical function and quality of life among elderly disaster victims. Applied Nursing Research.

[B12-behavsci-16-00503] Hashimoto A., Miyazaki M., Ishimaru M. (2015). Adaptation of the elderly in shelters and temporary housing after the Great East Japan Earthquake. Health Emergency and Disaster Nursing.

[B13-behavsci-16-00503] Hikichi H., Aida J., Matsuyama Y., Tsuboya T., Kondo K., Kawachi I. (2020). Community-level social capital and cognitive decline after a natural disaster. Social Science & Medicine.

[B14-behavsci-16-00503] Keldal G. (2015). Warwick-Edinburgh Mental İyi Oluş Ölçeği’nin Türkçe Formu: Geçerlik ve Güvenirlik Çalışması. Journal of Happiness and Well-Being.

[B15-behavsci-16-00503] Kwak Y., Chung H., Kim Y. (2017). Differences in health-related quality of life and mental health by living arrangement among Korean elderly. Iranian Journal of Public Health.

[B16-behavsci-16-00503] Lee J., Aldrich D. P., Kiyota E., Tanaka Y., Sawada Y. (2022). Social capital building interventions and self-reported post-disaster recovery in Ofunato, Japan. Scientific Reports.

[B17-behavsci-16-00503] Ozgun-Basibuyuk G., Kaleli I., Efe M., Tiryaki F., Ulusal F. B., Demirdas B., Dere B., Ozgur O., Koc O., Tufan I. (2021). Depression tendency caused by social isolation: An assessment on older adults in Turkey. Advances in Gerontology.

[B18-behavsci-16-00503] Sasaki Y., Tsuji T., Koyama S., Tani Y., Saito T., Kondo K., Kawachi I., Aida J. (2020). Neighborhood ties reduced depressive symptoms in older disaster survivors: The Iwanuma Study. International Journal of Environmental Research and Public Health.

[B19-behavsci-16-00503] Sevim Y. (2024). Deprem nedeniyle zorunlu göçe maruz kalan yaşlı bireyler üzerine sosyolojik bir araştırma. Kent Akademisi.

[B20-behavsci-16-00503] Shenk D., Mahon J., Kalaw K. J., Ramos B., Tufan I. (2010). Understanding the disaster experience of older adults by gender: The experience of survivors of the 2007 earthquake in Peru. Health Care for Women International.

[B21-behavsci-16-00503] Tennant R., Hiller L., Fishwick R., Platt S., Joseph S., Weich S., Parkinson J., Secker J., Stewart-Brown S. (2007). The Warwick-Edinburgh Mental Well-Being Scale (WEMWBS). Health and Quality of Life Outcomes.

[B22-behavsci-16-00503] Toguc H. (2025). Health effects of plant-based diets in university life. Iranian Journal of Public Health.

[B23-behavsci-16-00503] Yavuz C., Bolukbasi S., Tekin N., Sahin S., Akcicek F. (2024). The role of older adults in psychological recovery after natural disasters. Natural disasters and older adults.

[B24-behavsci-16-00503] Yotsui M., Campbell C., Honma T. (2016). Collective action by older people in natural disasters: The Great East Japan Earthquake. Ageing and Society.

[B25-behavsci-16-00503] Zimet G. D., Dahlem N. W., Zimet S. G., Farley G. K. (1988). The multidimensional scale of perceived social support. Journal of Personality Assessment.

